# The epidemiological and clinical profile of COVID-19 in children: Moroccan experience of the Cheikh Khalifa University Center

**DOI:** 10.11604/pamj.supp.2020.35.2.23571

**Published:** 2020-06-01

**Authors:** Nabila Chekhlabi, Chafik El Kettani, Amal Haoudar, Abdelkrim Bahlaoui, Mohamed Mahi, Said Ettair, Nezha Dini

**Affiliations:** 1 Department of Paediatrics, Cheikh Khalifa´s Hospital, Casablanca, Morocco; 2 The International University Hospital Cheikh Khalifa Ibn Zayed, Casablanca, Morocco; 3 Mohammed VI University of Health Sciences UM6SS, Casablanca, Morocco; 4 Department of Intensive Care Unit, Cheikh Khalifa´s Hospital, Casablanca, Morocco; 5 Department of Respiratory Diseases, Cheikh Khalifa´s Hospital, Casablanca, Morocco; 6 Department of Radiology, Cheikh Khalifa´s Hospital, Casablanca, Morocco; 7 Faculty of Medicine and Pharmacy of Rabat, Mohamed V University, Rabat, Morocco

**Keywords:** COVID-19, children, coronavirus

## Abstract

**Introduction:**

COVID-19 is an infectious disease caused by a new coronavirus. The first cases were identified in Wuhan. It rapidly spread causing a pandemic worldwide. The incidence and severity of this disease are likely to be different in children compared with adults. Few publications of COVID-19 in children have been published. Our Moroccan paediatric series is among the first studies on this disease in Africa.

**Methods:**

We included all children with COVID-19 who were admitted and treated at the hospital from March 25 to April 26, 2020. We have collected information, including demographic data, symptoms, imaging data, laboratory results, treatments and clinical progress from patients with COVID-19.

**Results:**

Since the outbreak of 2019 novel coronavirus infection (2019-nCoV) in Morocco, a total of 145 COVID-19 confirmed cases have been reported in the Cheikh Khalifa’s Hospital. Among this cases, 15 children were registered. The median age of patients was 13 years. There were 7 boys and 8 girls. Five children are asymptomatic, 8 have mild symptoms and 2 have a moderate respiratory difficulty. The RT-PCR test results were positive in all patients. Radiologically, we found in 2 cases, multiple nodules with ground-glass opacities on the chest scan. The treatment was based on the combination of hydroxychloroquine and azithromycin. Evolution under treatment was good for all patients.

**Conclusion:**

This study describes the profile of COVID-19 in child in a Moroccan hospital and confirms that the severity of illness in children with COVID-19 to be far less than adults.

## Introduction

The world is currently experiencing a pandemic of infectious disease called COVID-19 (coronavirus disease 2019), caused by a new virus coronavirus: SARS-CoV-2. Coronaviruses are a large family of enveloped RNA viruses that cause illnesses ranging from a common cold to more severe diseases such as MERS or SARS. The first cases in Wuhan, China, did not involve children, which suggested that the disease was not symptomatic in children [1]. Now, that the outbreak is global with more than 3.3 million confirmed cases and 238 000 deaths (as of May 01, 2020) [2], we can evaluate more accurately the epidemiological profile of this disease in children. The Korean center for disease control and prevention reported that, up to 20 March, 6.3% of all cases that tested positive for COVID-19 were children under 19 years of age [3]. Worldwide, the confirmed cases were mostly among adults, with paediatric cases rarely reported [4]. Therefore, little is known about the clinical profile of infected children around the world. Here, we report our experience, among the first studies in Africa, detailing the epidemiological and clinical profile in children with COVID-19 in University Moroccan Hospital. Morocco has identified until April 26, according to the direction of epidemiology and fight against diseases, about 4900 cases of confirmed COVID-19 including more than 390 paediatric cases [5]. In Casablanca, the economic capital of the country and according to the same source, there have been 97 cases of children affected with a cumulative incidence of 4.8 per 100,000 children [5].

**Context:** Cheikh Khalifa hospital (CKH) is an international university and multidisciplinary center. Since the start of this pandemic in Morocco, the ministry of health has designated several hospitals to handle COVID-19 cases, including the CKH. There was a rapid increase in the disease with the reception and care of many affected families in our hospital, then there was a gradual and regular decrease. From the first confirmed case of children admitted to our structure, we conducted a retrospective study focused on different characteristics of this category. All the hospitalised children received the same therapeutic protocol, codified by the ministry of health and generalized to all the country.

## Methods

In order to analyse the COVID-19 disease in children, we conducted a retrospective study and we included all children hospitalised for COVID-19 during a period of 1 month (from March 25 to April 26, 2020) within the Hospital International University Cheikh Khalifa of Casablanca city in Morocco. Children and adolescents were defined as being under 19 years old. Demographic information and all characteristics including exposure, medical history, symptoms, chest computed tomographic (CT) scan, laboratory tests, treatments and evolution of each patient were obtained from the electronic medical record system of Cheikh Khalifa´s Hospital. Suspected cases were identified if a child had 2 of the 4 following conditions: close contact with a confirmed case within the 2 last weeks; fever and or respiratory signs (cough, difficulty breathing, anosmia) and or digestive symptoms (vomiting, diarrhea) and or asthenia, myalgia; abnormal laboratory test (white blood cell count, lymphocyte count, level of C-reactive protein procalcitonin, lactate dehydrogenase, ferritin and D-dimer); or abnormal chest radiograph imaging result. COVID-19 was confirmed in all patients with positive results in the real time reverse transcription polymerase chain reaction (RT-PCR) test on nasopharyngeal swab specimens. The exclusion criteria are all cases who are over 19 years of age and/or do not meet the previously detailed criteria for confirmation of the disease. The inclusion criteria are: age under 19; ≥ 2 of the above 4 conditions of suspicion of COVID-19; positive RT-PCR test on nasopharyngeal swab specimens. All confirmed children were hospitalised and treated with regular check-ups. The treatment given, in symptomatic children, is codified nationally by the ministry of health. Patients are declared cured if they are doing well clinically with 2 negative RT-PCRs. All ethical principles were considered in this article. The participants were informed about the purpose of the research and its implementation stages; they were also assured about the confidentiality of their information.

## Results

Since the outbreak of the new coronavirus infection 2019 (COVID-19) in Morocco, from March 25 to April 26, 2020, more than 400 suspected cases have been admitted to Cheikh Khalifa University Hospital. Of the 145 laboratory-confirmed cases, 15 children were reported with a percentage of 10.3%. The median age of patients was 13 years (interquartile range: 5 - 19 years). There were 7 boys and 8 girls (sex ration: 0.87). All children were Moroccan, coming from the Casablanca city and had family exposure. All children were vaccinated according to national vaccination program including tuberculosis vaccine. Among these children, two had uncontrolled asthma and one has allergic rhinitis. The average consultation time after onset of symptoms is 3 days (extremes 1 and 7 days). Regarding the severity of confirmed cases, 5 (33.4%), 8 (53.4%) and 2 (13.2%) cases were diagnosed as asymptomatic, mild and moderate respectively. Among symptomatic children, 9 had fever and dry cough with moderate breathing difficulty in 2 cases, 2 had watery diarrhea and 3 had anosmia (Table 1). The 2 children who were respiratory difficulty had sibilant rales at auscultation and had saturation between 92 and 95%. The RT-PCR test results were positive in all patients. In all cases, the values of CRP, procalcitonin, white blood cells, lactate dehydrogenase, kidney and liver function were normal.

**Table 1:** Table summarizing the clinical symptoms of the 10 symptomatic patients

Patients	Age (Y)	Sex	Fever	Cough	Dyspnea	Myalgia	Anosmia	Diarrhea
Patient 1	5	Male	Yes	Yes	Yes	No	No	No
Patient 2	12	Female	Yes	Yes	No	Yes	Yes	No
Patient 3	10	Female	Yes	Yes	No	Yes	No	Yes
Patient 4	12	Female	Yes	Yes	No	Yes	No	No
Patient 5	14	Male	Yes	Yes	No	No	No	No
Patient 6	10	Female	Yes	Yes	No	Yes	No	No
Patient 7	15	Male	Yes	Yes	Yes	Yes	No	Yes
Patient 8	12	Female	No	No	No	Yes	Yes	No
Patient 9	18	Male	Yes	Yes	No	Yes	Yes	No
Patient 10	18	Female	Yes	Yes	No	Yes	No	No

**Figure 1:**
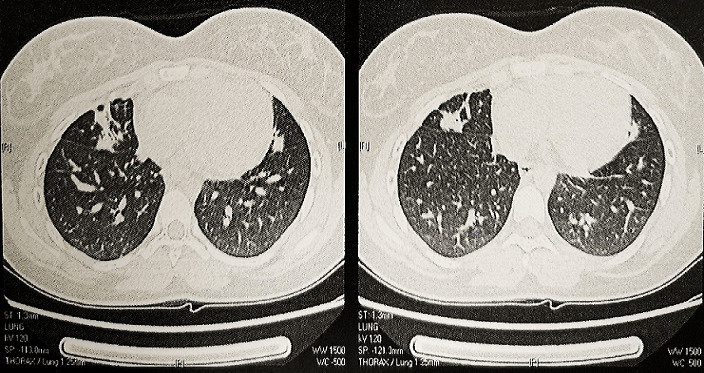
Chest CT scan in axial sections showing bilateral multiple nodules with ground-glass opacities (patient 2)

**Figure 2:**
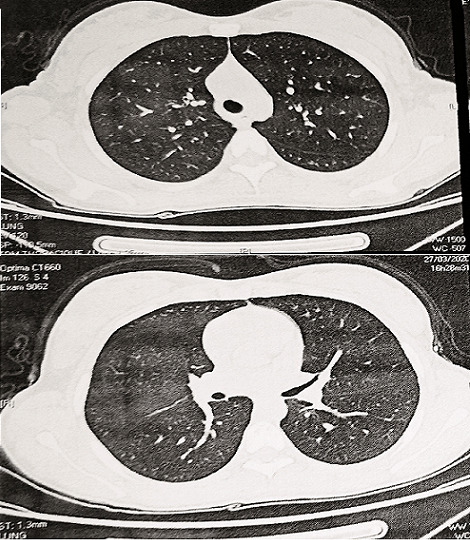
Chest CT scan in axial sections noting ground-glass opacities (patient 10)

The D-dimer levels were high in 3 patients (greater than 500μg/l). Radiologically, we found in 2 cases, multiple nodules with ground-glass opacities in both lungs on the chest CT scan (Figure 1, Figure 2). Treatment modalities were focused on symptomatic and specific support. Symptomatic treatment was based on bed rest, vitamin C and paracetamol if fever. Beta 2 agonist inhaler with a valved chamber and oral corticoids were administered in the 2 asthmatic children. Low molecular weight heparin was used in adolescents with high D dimers levels. We adopted, following the ministerial recommendations, hydroxychloroquine-based therapy preceded by an electrocardiogram in search of heart rhythm disorder. All our symptomatic patients received bi-therapy based on hydroxychloroquine and azithromycin at the respective dose of 10mg/Kg/Day for 10 days and 20mg/Kg/Day for 7 days. No children received antivirals. The response to treatment was good for all patients. No side effects were noted. Until April 26, 2020, two patients are stable and still hospitalized in the paediatric service under supervision, the other thirteen have recovered clinically. Their RT-PCRs performed at two days´ interval was negative and they returned home.

## Discussion

Since December, 2019, COVID-19 has spread rapidly around China and the world. The world health organization (WHO) has declared the infection a global pandemic. Nationwide case series of 2135 paediatric patients with COVID-19 reported to the chinese center for disease control and prevention from January 16, 2020, to February 8, 2020 were included [6]. The United States of America released a study, which looked at more than 2,500 coronavirus cases among children younger than 18 in the US between February 12 and April 2. That’s the largest research sample of children with the coronavirus to date. The data suggested that children are less likely to develop symptoms than adults. Of all reported cases, only 1.7% were children [7]. During the first 2 weeks of the epidemic in Madrid region (Spain), 41 of the 4695 confirmed cases (0.8%) were children younger than 18 years [8]. The reason for having a higher rate of infected children (10%) than other studies is the hospitalization of five sick families containing 2 or 3 confirmed children for each of the families. The COVID-19 incubation period is approximately 2 to 14 days (but most often between three to five days) and transmitted by respiratory droplets and close contact with an infected person. Since most children have been exposed to family members and/or other children with COVID-19, this clearly indicates person-to-person transmission [6].

To fight against human-to-human transmission and the spread of this pandemic, Morocco implemented rigorous preventive interventions very early on, such as home confinement, social isolation, school closings, compulsory wearing of masks and search for contacts. Although clinical manifestations of children´s COVID-19 cases were generally less severe than those of adult patients, young children, particularly infants and those with chronic disease were vulnerable to infection and were more likely to have severe clinical manifestations [9]. This means that children who do not have underlying conditions, such as chronic heart or lung disease, diabetes mellitus or immunosuppression (chemotherapy, high doses of corticosteroids), have a much lower risk of severe forms of COVID-19 than other age groups [10]. In our study, we did not find any severe or unstable paediatric case, unlike adults where there were about twenty serious cases with 14 deaths. We also found, in our series, that the 2 children with uncontrolled asthma presented a more telling respiratory symptoms than the others. However, we noted that atopy represents only 3 out of 15 patients (20%), which leads to a discussion on the real role of atopy in COVID-19.

Is atopy a risk factor for the seriousness of the disease or, on the contrary, does it play a preventive role because of the immune imbalance weighted towards the TH2 response instead of TH1? This hypothesis must be a research soon. The symptoms of COVID-19 are similar to those of a simple flu: infected children often have fever, dry cough and rarely difficulty breathing. Digestive symptoms (vomiting, diarrhea), asthenia and myalgia are also described in the different series. However, the infection can progress more seriously and lead to pneumonia, failure of several organs, severe acute respiratory syndrome, or even death in the most serious cases [11]. We present the findings of Dong et al. who reported in china, a series of 731 children with laboratory-confirmed COVID-19 disease, approximately 13% of cases had asymptomatic infection, 42% were mild, 40% were moderate (pneumonia without hypoxemia), only 5% of cases were severe (dyspnea and hypoxemia) and 0.6% progressed to acute respiratory distress syndrome (ARDS) or multiorgan dysfunction, a rate that is significaly lower than that seen in adults [11].

Regarding the results of our study, we have 33.4% of asymptomatic children and more than half of the cases had a mild form of the disease. We conclude that the children may play a major role in community-based viral transmission. Since the majority of available data suggest that children can be asymptomatic and have nasopharyngeal carriage, rather than lower respiratory tract infection [6]. There is also evidence of faecal shedding in the stool for several weeks after diagnosis [4,12]. Radiological semiology in children is still poorly described. The first publications describe lesions similar to those of adults, although less severe. They show ground glass under pleural uni or bilateral and in half of the cases, zones of consolidation with halo sign [13]. A recent study in China analysed the CT images of 15 children with COVID-19, 10 of whom were asymptomatic and 5 had mild symptoms. Among the 15 children, pulmonary inflammatory lesions were observed in 9. Nodular ground glass opacity was the most common finding and sub pleural patchy opacities were also observable, with all lesions limited to a single lung segment [14].

Chest CT is a highly sensitive diagnostic tool to detect pneumonia and the sensitivity for COVID-19 is reported to be 97.5% [15]. In our series, we had 2 pathological chest CT scanners with bilateral multiple ground glass opacity (Figure 1, Figure 2). The 1st case had a dry cough without dyspnea as long as the 2nd case was asymptomatic. The 2 cases which presented a moderate respiratory gene had a completely normal CT scan. Biologically, white blood cell count is normal or decreased, with decreased lymphocyte count; most patients display normal C-reactive protein and procalcitonin levels. Severe cases show high D-dimer levels and ferritin rate. Samples from nasopharyngeal swabs, sputum, lower respiratory tract secretions, stool and blood are tested positive for 2019-nCoV PCR [12,16]. We present in table comparative results between different series (Table 2) [4,14,17]. Some institutions have developed algorithms to determine when treatment with hydroxychloroquine or remdesivir may be warranted [11]. Children with underlying conditions or with immunocompromising treatment and children younger than three years appear to be at the greatest risk for progression of COVID-19 disease and therefore indication of the specific treatment mentioned above [11].

**Table 2:** Main comparative data between the series

Information	Our series (Morocco)	Rahimzadeh's series (Iran) (16)	Shanghai's series (China) (4)	Shenzhen's series (China) (17)
Number of cases	15	9	10	15
Median age (Y)	13	5	6	7
Sex (M/F)	7/8	6/3	4/10	5/10
Family contact	All	All	7/10	8/15
**Clinic symptoms**				
Asymptomatic	5	No	4	8
Fever, cough	9	All	6	7
**Biology**				
RT- PCR positive	All	3/9	All	All
Lymphopénie	3	3	3	8
Chest CT scan abnormalities	3 cases	9 cases	4 cases	9 cases
Intensive care unit	No	2	No	No

In an Iranian series (9 cases), most of patients were treated with administrating oxygen, nebulizing with β2-agonist, using probabilistic antibiotics, hydroxychloroquine and oseltamivir [17]. Sometimes antibiotic treatment of bacterial superinfection may be necessary. According to the ministerial and national therapeutic protocol, we put symptomatic children under the association hydroxychloroquine and azithromycin with adapted paediatric dosages. Hydroxychloroquine, an antimalarial drug, known to have anti-inflammatory and antiviral effects, with low cost and easy availability, is a promising practice to treat COVID-19 infection under reasonable managements. One of the major French studies suggested that hydroxychloroquine, in combination with the antibiotic azithromycin, could work as a treatment for COVID-19 [18]. The results of this study should not be conclusive given its observational design. According to a more recent American study, the administration of hydroxychloroquine in patients with COVID-19 was not associated with a significantly reduced risk of complication or death [19].

Randomized controlled trials of hydroxychloroquine efficacy in patients with COVID-19 are needed. The Indian medical research council (IMRC) recommended the use of hydroxychloroquine as a prophylactic against COVID-19 in health care workers and asymptomatic contacts of laboratory-confirmed cases of COVID [20]. Why not follow this prophylactic practice among health professionals and fragile people to protect them now and in the future from other seasonal outbreaks? The number of COVID-19 cases in countries with fragile health systems is lower than expected [21]. Among the hypotheses mentioned, it is that the tuberculosis vaccine could have a protective effect against the coronavirus, either by reducing the risk of being infected, or by limiting the severity of the symptoms [21]. Note that all our patients are vaccinated against tuberculosis. Until a specific vaccine is developed, COVID-19 vulnerable populations and health personnel could be immunized with BCG vaccines [21].

## Conclusion

To our knowledge, our study is one of the first paediatric series in Africa to describe the clinical, radiobiological and evolutionary characteristics of COVID-19 in children. the clinical presentation was mild to moderate without mortality. While the initial data reported so far on children is reassuring, paediatricians need to continually update their knowledge and be aware of the risks, especially in infants and children with comorbidities.

### What is known about this topic

- COVID-19 is particularly rare and less severe in immunocompetent children than in adults;
- Few studies (mainly among Asian and European populations) have been published on COVID-19 in this age group.

### What this study adds

- The literature on COVID-19 is developing day by day all over the world and our African series would contribute to increase knowledge;
- This study helps to identify the clinical characteristics and the management of COVID-19 in Moroccan children.

## Competing interests

The author declares no competing interests.
